# Gastric Cancer: Bibliometric Analysis of Epidemiological, Geographical and Socio-Economic Parameters of the Global Research Landscape

**DOI:** 10.34172/ijhpm.2020.29

**Published:** 2020-03-01

**Authors:** Doris Klingelhöfer, Markus Braun, Norman Schöffel, Dörthe Brüggmann, David A. Groneberg

**Affiliations:** Institute of Occupational, Social and Environmental Medicine, Goethe University, Frankfurt, Germany.

**Keywords:** Stomach Cancer, Cancer Epidemiology, Research Funding, Bibliometric Analysis, Socio-Economic Influences

## Abstract

**Background:** Although the incidence and mortality of gastric cancer (GC) decreased in the last years, some countries remain highly affected. Especially in high-income economies the cases of cardia types are steadily increasing. Currently, GC is ranked third as cause for cancer death worldwide, whereby two-thirds of deaths still occur in low-income countries. But the global numbers are changing, and new regional challenges must be addressed.

**Methods:** Therefore, this disease has been chosen for in-depth bibliometric analyses that combines the evaluation of publication meta-data with density equalizing visualization techniques. This study focuses on the chronological and geographical characteristics of GC research worldwide. Epidemiological and socio-economic parameters were analyzed and the influence of political framework conditions was examined. In addition, international collaborations and research priorities were evaluated.

**Results:** In the last years, the publication numbers are rising more extensively in comparison to other cancer types. Albeit the usual leading positions, the United States is not the most publishing country on GC. It occupies the third position. Instead, China and Japan are the most publishing countries and together with South Korea also the key players as well as the most affected countries. These countries’ governments are also the main donors for GC-research. The number of articles and the expenditures for research and development (R&D) as well as the incidence rate are correlated. Despite the scarce contribution of low-economic countries to the publication output, international collaborations lead to a modest output in those regions.

**Conclusion:** This study pools background information for scientists, practitioners, funders and decision-makers by providing information on the development and priorities of GC research. Adaptive international approaches and partnerships are crucial to meet future changing epidemiological features worldwide.

## Background

Gastric cancer (GC) is a heterogeneous disease, determined by specific genetic modifications in conjunction with the occurrence of various environmental risk factors. Here, nutrition, life style, occupational exposures and technological development status has to be named among others.

After breast, prostate, lung, colorectal, and cervix cancer, GC is assessed to be the neoplasm with the sixth highest incidence rate in 2018.^1^ Affecting 723 000 deaths in 2012, GC was ranked third as cancer cause of deaths worldwide.^2^ The incidence rates vary strongly and differ up to 20-fold in various regions.^3,4^ Low-income countries are mostly affected. Additionally, approximately two-thirds of the deaths caused by GC (75.79%) occur in low-income countries. The highest mortality rates are presumed for Eastern Asia, where the most of the cases (405 000 from 952 000) occur in China alone.^5^ But also, Eastern Europe and Central and South America are high incidence regions, while the risk of disease is relatively low in North America, Southern Asia, Australia, and in parts of Africa. Worldwide, men are still diagnosed more than twice as often as women.^1^

The differing histology between cardia and non-cardia malignancies lead to extremely varying epidemiological backgrounds. Though the incidence rates of non-cardia GC decreased in the last decades particularly in high-income countries, associated with the declining infection rate of *Helicobacter pylori*, the rates of gastric corpus cancer were found to be increasing.^6^ This type is defined as corpus-, young age- and female-dominant, therefore called CYF-cancer.^7^ Like esophageal adenocarcinoma, that is currently gravely spreading in the industrial world, this newly observed type of GC appears to be increasing in an equal way.^6-8^ Therefore, a completely new epidemiological direction for GC can be assumed in the future. It is to be expected that the identification of this new cancer type will initiate new regional approaches leading to a changing of the global research landscape, which will have to be assessed by a future analysis that can be compared to the current findings.

There already exists a huge basis of oncological, clinical, radiological, and epidemiological knowledge on GC, resulting from profound global research. Therefore, this study aims at finding answers to the questions: What have been the most decisive driving forces to engage in GC research until now? What are the big players, and most of all, are the global research efforts adequate for the evaluation of varying risk factors or incidence rates? How is the global research landscape composed? Therefore, this study provides an in-depth bibliometric analysis of the publication output on GC from chronological, geographical, epidemiological, and socio-economic perspectives.

The study is embedded in a methodological platform that pools analyses of important medical issues into an information source for scientists, practitioners, funders, and decision-makers. The findings can help to plan and establish a global network that includes high infrastructure countries, most affected and less developed regions alike to satisfy the globally differing backgrounds, needs and possibilities.

## Methods

### Methodological Platform

This study is methodologically incorporated in the bibliometric platform NewQIS (New Quality and Quantity Indices in Science).^9^ Within its scope various analyses on different biomedical issues were already carried out.^10-12^ The established methodology combines advanced bibliometric approaches with state-of-the art visualization techniques. Its data source is the Core Collection of Web of Science (WoS) that is one of the most extensive and highly esteemed scientific databases that sets high requirements for its listed journals. Additionally, it provides the Journal Citation Reports (JCR) that allows the analysis of citation numbers. Only a few other data bases offer citation numbers, albeit with the disadvantages of covering smaller time periods or insufficient quality requirements.^13^

### Search Procedure and Data Processing

The following search term was applied in the title search of WoS: (*stomach* OR *gastric*) AND (*neoplasm* OR *cancer* OR *carcinoma*). The title search secured a representative data pool with only a minimum of faulty entries. By filtering the document types, only original articles were incorporated in the data base to relate the analysis to the research activities.

The meta-data of the results were downloaded and sorted according to the included tags that represent an information unit. They were stored in a MS Access database and sorted according to the evaluation parameters.

### Performed Analyses

The retrieved data was analyzed according to their chronological and geographical features, eg, publication numbers, research areas. Additionally, socio-economic^14^ and epidemiological data^15^ was evaluated to assess the performance of the publishing countries in a meaningful way. Ergo, the scientific performances of the Organization for Economic Co-operation and Development (OECD) member countries were analyzed by determination of a possible association between the number of articles and the incidence rates of GC^16^ as well as the expenditures for Research and Development (R&D).^17^ Different citation parameters served for assessment of the relevance of the retrieved articles. So, big players could be identified and incentives and benchmarks for research determined and assessed.

Another point was the identification of the funding institutions, respectively programs that mostly supported global research on GC. For this purpose, the funders were specified by the indications of the authors. All available data was retrieved software supported. Due to the wide variety of funder notations, data had to be unified manually. This was done by grouping them to the countries of origin and the type of funding. Subsequently, unification of funders could be performed. WoS provides data on funding from 2008 onwards, so that the respective results are limited to the last decade.

For comparison purposes, the development of publication numbers of esophageal, cervix, and endometrial cancer were chosen. On the one hand, a related cancer type and on the other hand, two cancer types out of another medical specialty were selected to generate a similar as well as a different comparison background. For this purpose, the times frame from 1900 to 2016 was defined to allow comparison with the results on the publication output on esophageal cancer, which was also analyzed within this time frame. The numbers on esophageal cancer have already been published.^18^

### Visualization of Findings

Density equalizing map projections by Gastner and Newman^19^ were applied to visualize the geographical findings. By means of this expressive method, the world map is distorted based on the value of an analysis parameter according to the osmotic density equilibrium. A huge value amplifies the size of the publishing country and low values downsize it. Consequently, a new picture of the world map emerges.

The VOSviewer technology was applied for visualizing the findings of the cluster analyses of keywords.^20^

## Results

From 1900 until 2017, a total amount of 34 194 articles (n) could be identified and included in the data base.

### Chronological Analyses

Descriptions of GC can be found in papyri from Egyptian antiquity, already in 3000 BC, indicating that stomach cancer has already been known since then. Not surprisingly, the first articles of this study on GC were published in the first evaluation year of 1900, too. But only after the Second World War the publication numbers reached middle double-digit values. The subsequent numbers developed exponentially until the maximum of n = 2698 articles was reached in 2016. This course is following a conventional pattern of scientific output.^21^

**Figure 1:**
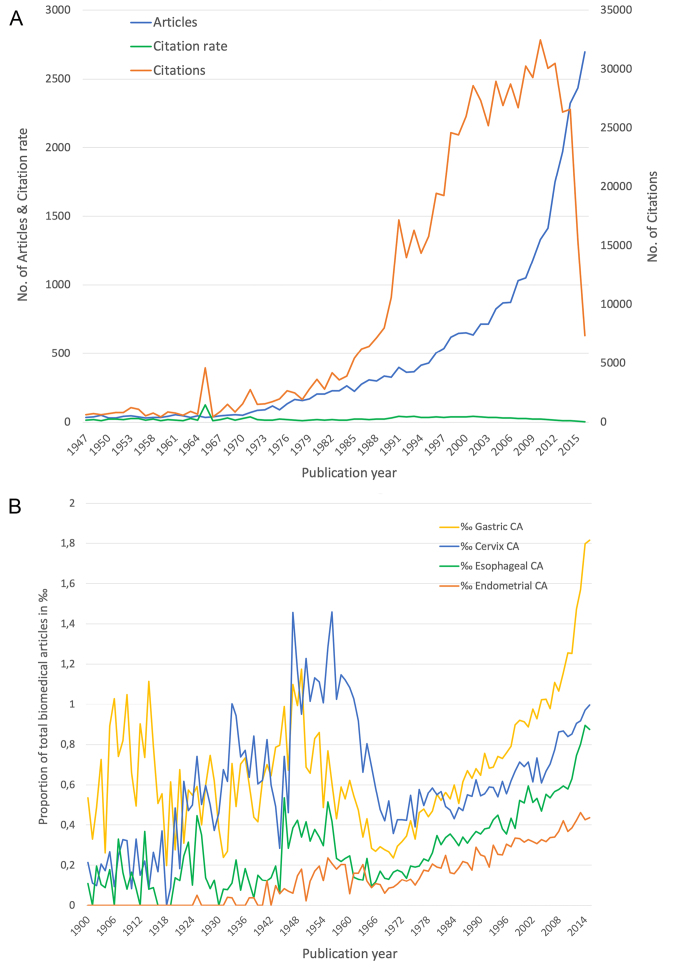
Development Over Time. (A) Number of articles, number of citations and citation rate of articles on GC from 1947 until 2016 (only years with >30 articles and completed years included). (B) Relative share of different cancer types of the overall biomedical output. Abbreviations: CA, cancer; GC, gastric cancer.

The development of the numbers of citations (c) showed also a significant increase over time that was more outstanding at the beginning of the 1980s. Salient peaks can be noticed in 1965 with c = 4590, 1991 with c = 17 163 and 2001 with c = 28 627. The articles published in 2008 received the highest amount of citations (c = 30 260). Articles that were published later have had little time to receive the full attention of the scientific community (Cited Half Life), so the citation numbers dropped rapidly to c = 7368. Looking at the average rate of citations per article (cr), the year 1965 presented itself outstanding, too (cr = 127.5) (Figure 1A).

The continuous course of the absolute publication numbers does not reflect the trend of the relative share of GC articles out of the totality of biomedical publications (Figure 1B).

With surpassing proportions at the beginning of the 20th century, it decreased in the 1960s. After that the numbers increased steadily. Compared to research on other cancer types, the publication numbers on GC increased exceptionally since the 1970s revealing a heightened interest.

Compared with the publication years of the most prolific articles, an association with the history of publication output can be stated. The years 1965, 1991, and 2001 are remarkable again (Table 1). The most cited article was published in 1965 and received 3836 citations. The Finnish article from Lauren is focused on the histological classification of GC for clinicians.^22^ In particular, the association with *H. pylori* infection was represented among the most cited articles.^22-25^ Articles on the influence of interleukin polymorphism^26^ as well as epidemiological studies were also often cited.^27,28^ The USA first-authored 7 of the 10 most cited articles. In addition, Finland, Japan, and South Korea published respectively one article as first-author-country.

**Table 1:** Most Prolific Articles on GC

**Country**	**Authors**	**Year**	**Citations**	**Title**	**Journal**
Finland	Lauren^22^	1965	3836	The two histological main types of gastric carcinoma: diffuse and so-called intestinal-type carcinoma: an attempt at a histo-clinical classification	*Acta Pathologica et Microbiologica Scandinavia*
USA	Parsonnet et al^23^	1991	3040	*Helicobacter pylori* infection and the risk of gastric carcinoma	*New England Journal of Medicine*
Japan	Uemura et al^29^	2001	2328	Helicobacter pylori infection and the development of gastric cancer	*New England Journal of Medicine*
USA	Correa^27^	1992	1879	Human gastric carcinogenesis: a multistep and multifactorial process--first American Cancer Society award lecture on cancer epidemiology and prevention	*Cancer Research*
USA	Macdonald et al^30^	2001	1868	Chemoradiotherapy after surgery compared with surgery alone for adenocarcinoma of the stomach or gastroesophageal junction	*New England Journal of Medicine*
USA, UK, Poland	El-Omar et al^26^	2000	1541	Interleukin-1 polymorphisms associated with increased risk of gastric cancer	*Nature*
USA	Nomura et al^24^	1991	1527	Helicobacter-pylori infection and gastric-carcinoma among Japanese-Americans in Hawaii	*New England Journal of Medicine*
USA	Devesa et al^28^	1998	1500	Changing patterns in the incidence of esophageal and gastric carcinoma in the United States	*Cancer*
South Korea, Belgium, Switzerland, China, Japan, Germany, Italy, Russia, Australia	Bang et al^31^	2010	1314	Trastuzumab in combination with chemotherapy versus chemotherapy alone for treatment of HER2-positive advanced gastric or gastro-oesophageal junction cancer (ToGA): a phase 3, open-label, randomised controlled trial	*The Lancet*
USA	Blaser et al^25^	1995	1174	Infection with helicobacter-pylori strains possessing CAGA is associated with an increased risk of developing adenocarcinoma of the stomach	*Cancer Research*

Abbreviation: GC, gastric cancer.

### Geographical Analyses

The provision of the author’s countries of origin in WoS from 1972 onwards allows the assignment of n = 23 604 articles reflecting 69% of the overall data base.

**Figure 2:**
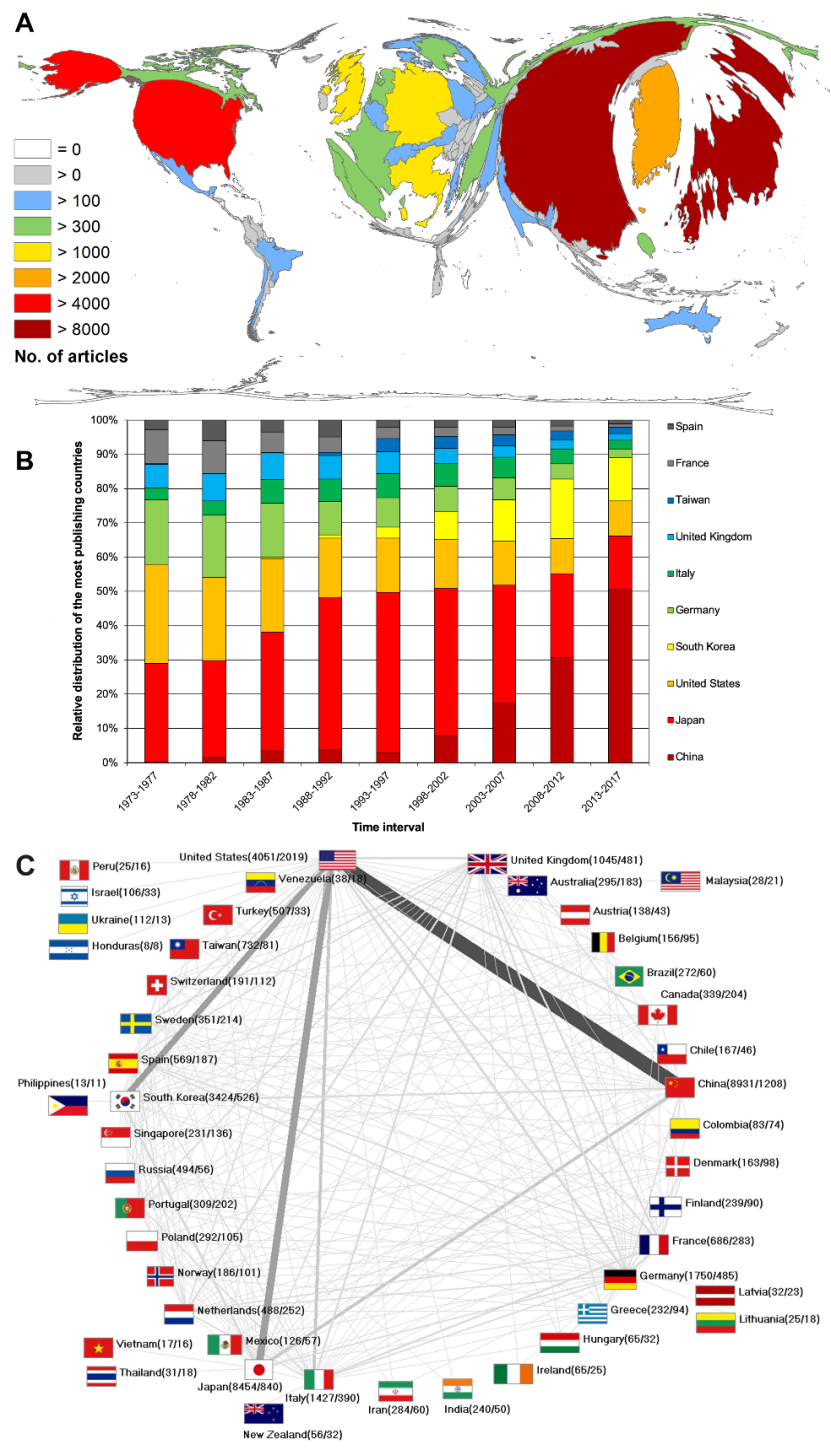
Global publication output on GC. (A) Number of articles per country. (B) Development of the relative share of the most publishing countries in 5-year intervals. (C) International network, numbers in brackets (number of publications/numbers of collaboration articles). Abbreviation: GC, gastric cancer.

Previous analyses on the publication output on gastroenterological or oncological issues^32,33^ have shown the dominance of the USA within the global research landscape. This deviated considerably form the result of the publication output on GC, where other countries were the big players (Figure 2A). The most publishing country was China with n = 8931 articles, followed by Japan on rank 2 with n = 8454 articles. On the third position, the USA followed with less than half of number of articles (n = 4051). South Korea reached rank 4 (n = 3424), followed by Germany (n = 1750). Another usually high-performance country, the UK, only ranked seventh (n = 1045) behind Italy (n = 1427).

The analysis of the development over time showed the increase of the Chinese publication share among the 10 most publishing countries starting with 0.26% and gradually growing to 50.54% until the last time interval (Figure 2B). South Korea started to publish on GC in 1985 (0.55% in the third interval 1983-1987) and raised its output to 12.55% between 2013 and 2017. In contrast, Japan’s contribution to articles on GC of the 10 most publishing countries showed the highest share between 1993 and 1997 (46.84%) and dropped to 15.71% in the last evaluation period step-by-step. The relative proportions of the USA and the leading European countries have decreased over time. For the whole period, the USA reduced its share from 28.97% to 10.15%, Germany from 18.72% to 2.56%, and the UK from 6.92% to 1.82%.

The publication output is reflected also in the collaborations between the countries (Figure 2C). The USA was the most collaborating country with n = 2019 articles that were worked out with other countries. Nearly 50% of its overall publications were collaboration articles. Here, the scientific linkage to China (n = 731), Japan (n = 360) and South Korea (n = 310) becomes visible.

**Figure 3:**
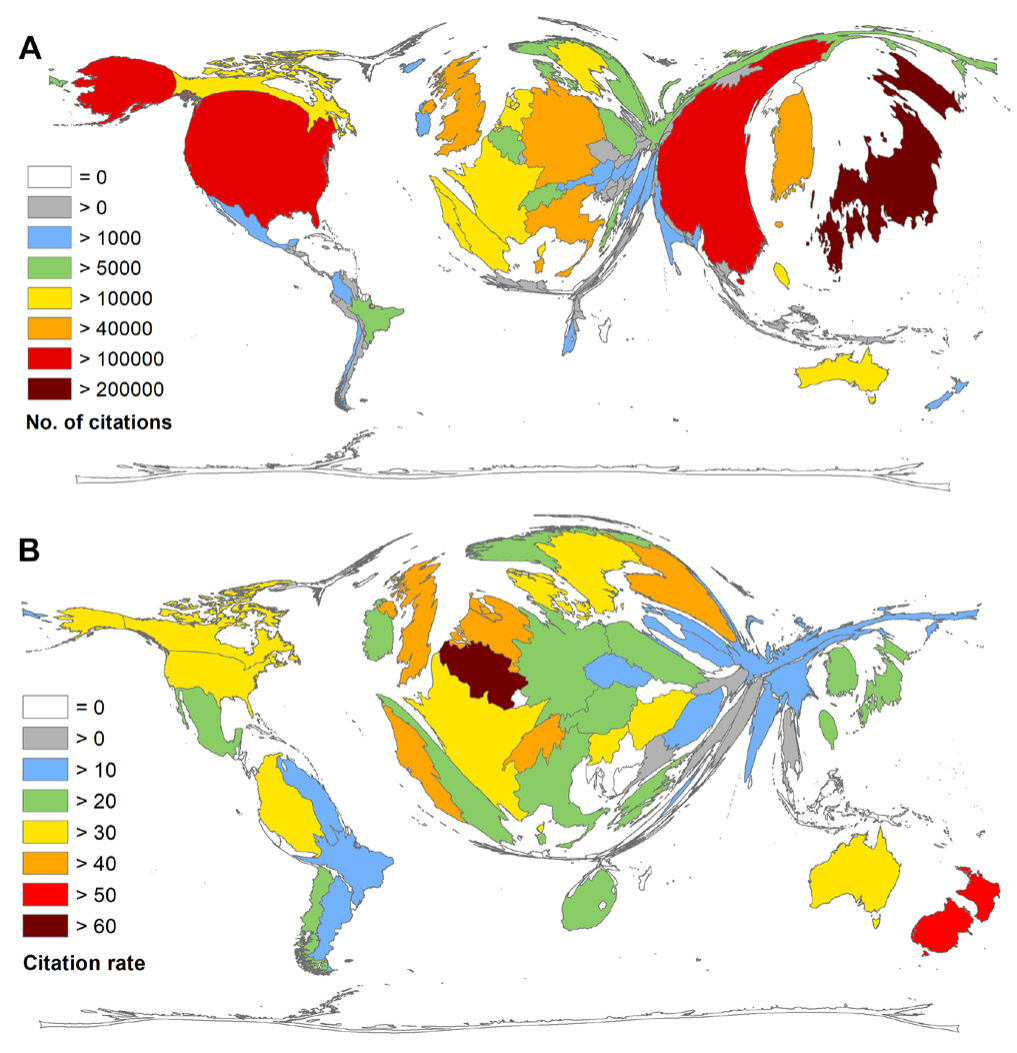
Global Citation Parameters of GC research. (A) Number of citations. (B) Average citation rate (threshold >30 articles). Abbreviation: GC, gastric cancer.

In terms of citation numbers, a slightly different picture became visible (Figure 3A). Here, Japan was the leading country with c = 228 969 received citations, followed by the USA (c = 154 648) and China (c = 120 978). South Korea reached rank 4 (c = 72 160), Germany rank 5 (c= 46 856), and the UK followed close behind (c = 45 631). The evaluation of the average citation rate (cr = number of citations/article) of the countries with more than 30 articles (threshold) emphasizes the contribution of Belgian scientists. Their 156 articles reached 9792 citations until now. Insofar, Belgium received the highest rate of this study with cr = 62.77. New Zealand followed on second position with cr = 55.95. The following order consists of Portugal (cr = 48.36), the Netherlands (cr = 47.36), the UK (cr = 43.67), and Switzerland (cr = 43.40). Japan was only ranked 30th (cr = 27.08) and Germany came short behind on rank 32 (cr = 26.78). Two South-American countries meeting the threshold came into view while assessing their citation rate: Colombia (cr = 35.23), and Chile (cr = 22.23) (Figure 3B).

**Figure 4:**
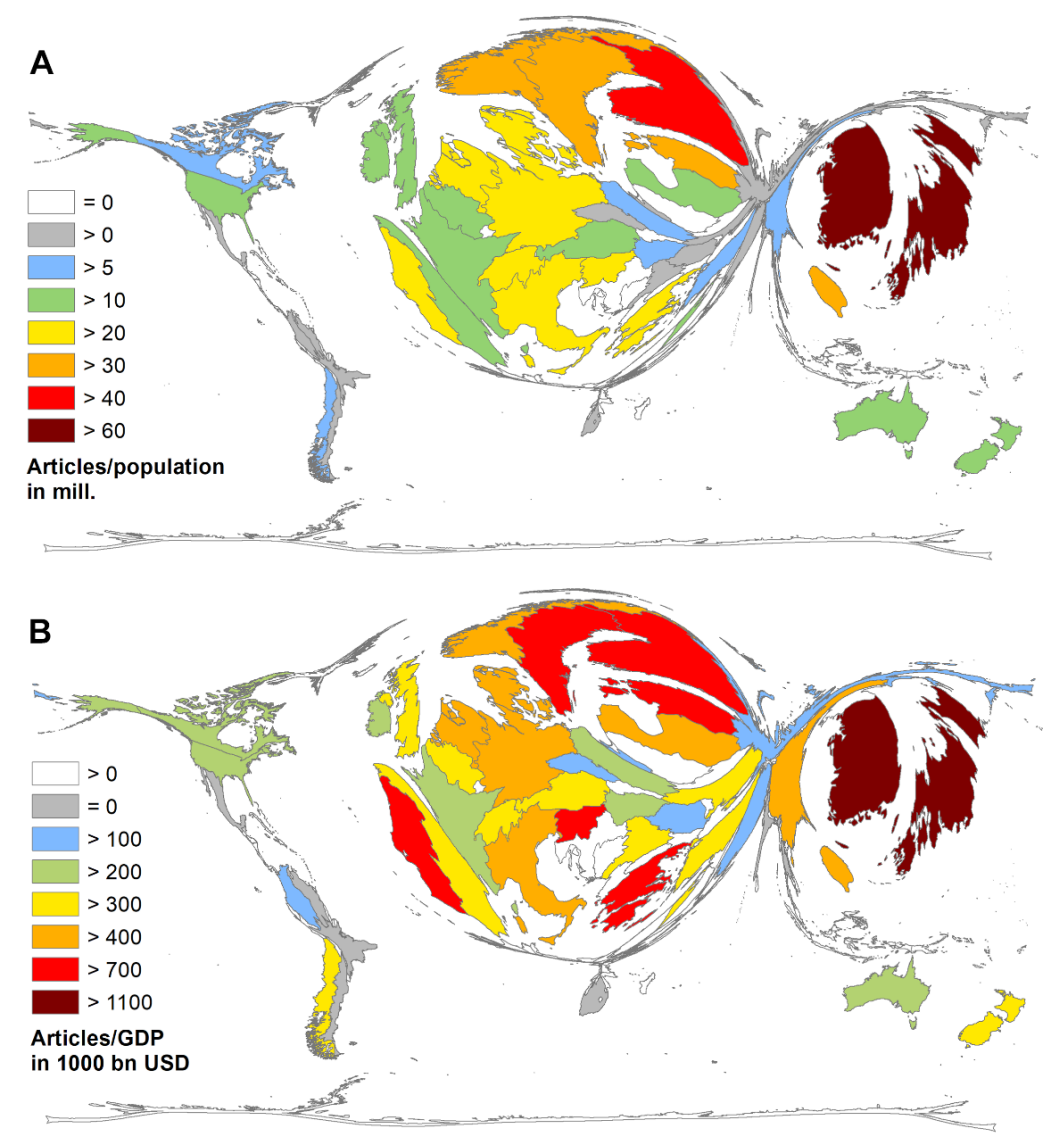
Socio-Economic Parameters of Global GC Research (Threshold >30 articles). (A) Ratio of the number of articles and the countries’ population in million inhabitants (R_POP_). (B) Ratio of the number of articles and the gross domestic product in US$1000 billion (R_GDP_). Abbreviation: GC, gastric cancer.

The inclusion of socio-economic aspects leads to a divergent picture of the global results once again (Figure 4; Supplementary file 1, Table S1). The first parameter sets the number of articles in relation to the population in million inhabitants of each country (R_POP_) (Figure 4A). Here, the leading countries were South Korea (R_POP_ = 67.24) and Japan (R_POP_ = 66.72). The subsequent order of the R_POP_ values is as follows: Finland (R_POP_ = 43.47), Singapore (R_POP_ = 39.95), and Sweden (R_POP_ = 35.52), to name the top five.

The analysis of the ratio between the number of articles and the gross domestic product of each country (R_GDP_) ranked South Korea first (R_GDP_ = 1775.01), followed by Japan R_GDP_ = 1714.11, Portugal (R_GDP_ = 1040.05), Finland (R_GDP_ = 999.16), and Estonia (R_GDP_ = 981.91) (Figure 4B).

Regarding both socio-economic parameters, the USA was dropped back with R_POP_ = 12.50 (rank 25) and R_GDP_ = 218.27 (rank 33).

### Correlation Analyses of Socio-Economic and Epidemiological Parameters

**Figure 5:**
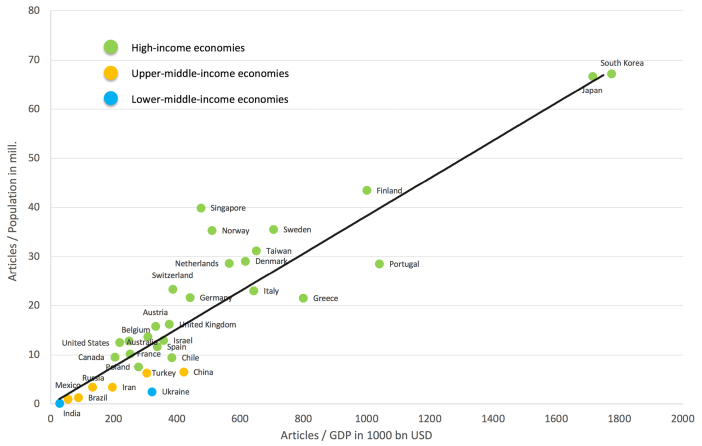
Correlation of the Socio-Economic Parameters: R_POP_ (number of articles/population in million) and R_GDP_ (number of articles/gross domestic product in US$1000 billion). Threshold = 30 articles per country. R^2^ = 0.84 (coefficient of determination); Spearman *** P < .0001.

Looking at the association between both socio-economic parameters, a significant correlation can be shown (r^2^ = 0.84, *P<*.0001). Moreover, the concentration of high-income countries on higher ranks and that of the middle-income countries at lower ranks could be illustrated (Figure 5). The classification of the economic status was retrieved from the grouping of the World Bank.^34^

**Figure 6:**
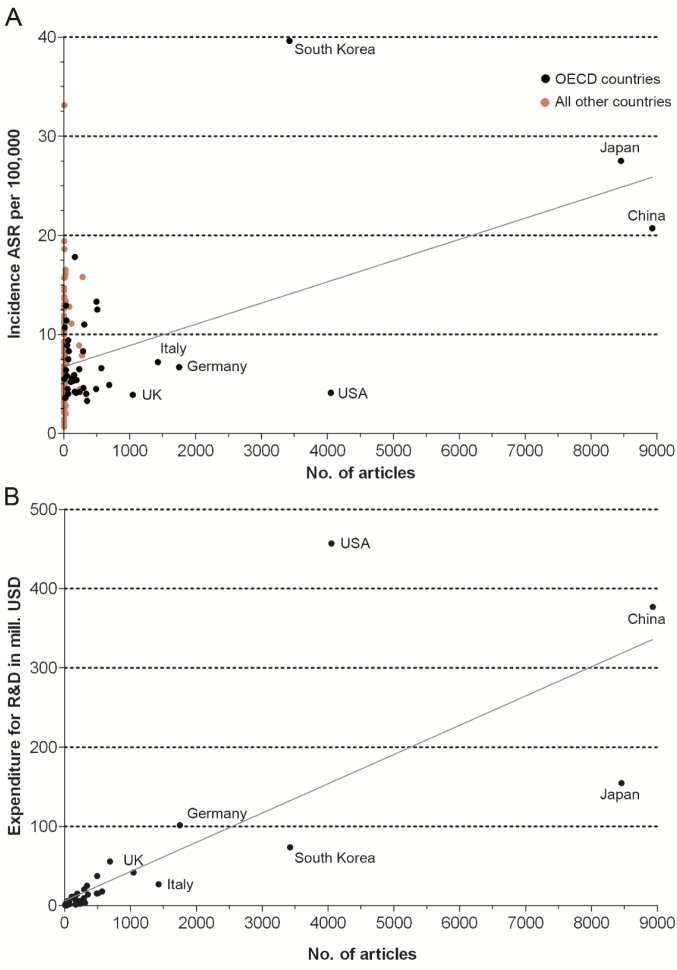
Incidence Rate^15^ and R&D Expenditures.^32^ (A) Number of articles and incidence rate ASR per 100 000. Brown dots: All countries: r^2^=0.15, (coefficient of determination), Pearson ***P < .0001. Black dots: OECD countries: linear regression r^2^=0.36, Pearson ***P < .0001. (B) Number of articles and expenditures for R&D in million US dollar for OECD countries: linear regression r^2^ = 0.63, Pearson ***P < .0001. Abbreviations: R&D, research and development; ASR, age standardized rate; OECD, Organization for Economic Co-operation and Development.

The analysis of the association between the incidence rate per 100 000 (ASR = age standardized rate) and the number of articles on GC showed a correlation (Pearson *P<*.0001) for OECD countries. In this context, it should be stressed that the highly affected East-Asian countries South Korea, China, Japan, and South Korea were among the most publishing countries, but non-OECD countries published relatively little irrespective of their incidence rates (Figure 6A). However, based on incidence rates of the International Agency for Research on Cancer (IARC) for 2018, a stronger correlation can be stated^15^ for all countries (Pearson *P<*.0001).

Moreover, for OECD countries, the correlation between the number of articles and the expenditures for R&D was highly significant. Despite this strong connection some countries occupy a more positive or more negative position. South Korea and Japan published comparatively more in relation to their overall R&D expenditures, whereas the USA contributed less compared with its overall R&D expenditures. Here, the relatively low epidemiological risk seems to play a role (Figure 6B) (Supplementary file 1, Table S2).

### Funding Analyses

**Figure 7:**
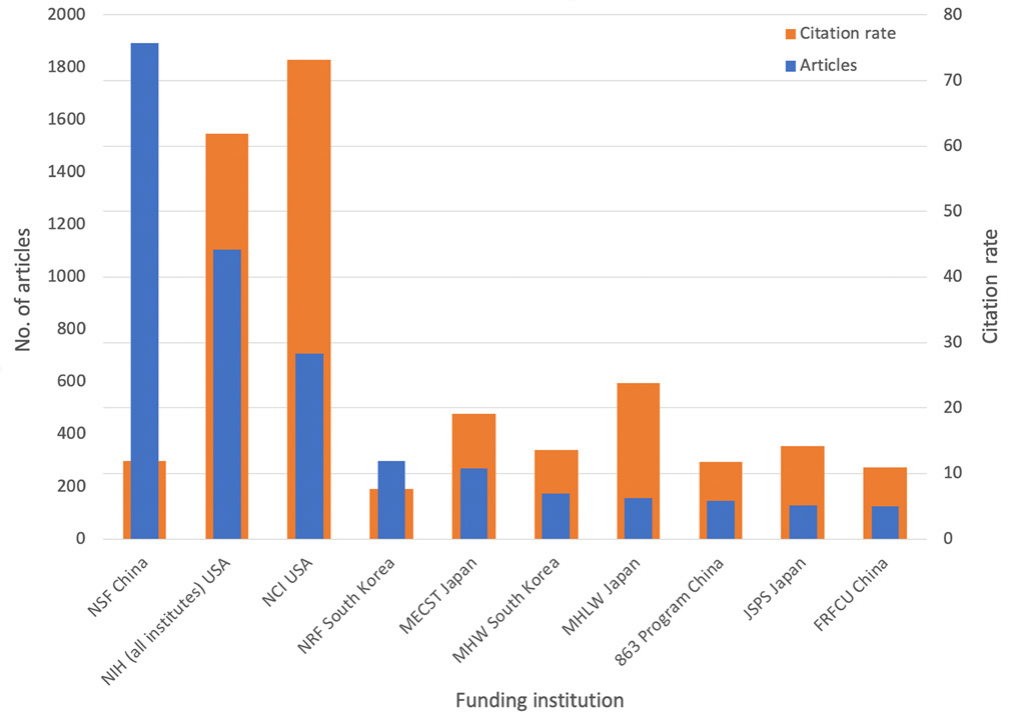
The Most Funding Institutions and Programs. Abbreviations: NSF, National Science Foundation; NIH, National Institutes of Health; NCI, National Cancer Institute; NRF, National Research Foundation; MECST, Ministry of Education, Culture, Science and Technology; MHW, Ministry of Health and Welfare; MHLW, Ministry of Health, Labor and Welfare; 836 Program, National High Technology Research and Development Program; JSPS, Japanese Society for the Promotion of Science; FRFCU, Fundamental Research Funds for the Central Universities.

The funding landscape for GC was mainly characterized by governmental grants from the most affected countries China, Japan, and South Korea (Figure 7). This is additionally supported by the strong correlation of publication numbers with the expenditures for R&D (Figure 6B). The government of USA, with the NIH (*National Institutes of Health*), came also into view on a leading position. The NIH is an authority of the *US Ministry of Health* and the largest organization for research funding worldwide. It reached the highest citation rate of the most funding institutions. As part of the NIH, the NCI (*National Cancer Institute*) coordinates the *National Cancer Program* that carries out cancer research and is listed separately, because it mostly contributed to this success and gained a higher citation rate on its own. This can be clearly assessed as high-impact research (Table 2). For the Asian countries it can be stated that each of the top Japanese grants reached more citations than its Chinese and South Korean counter parts.

**Table 2:** The Most Funding Programs and Institutions

**Country**	**Funder/Program**	**Grants**
China	National Science Foundation	1892
USA	NIH (all) USA	1103
USA	NCI USA	709
South Korea	National Research Foundation South Korea	298
Japan	Ministry of Educations, Culture, Science and Technology Japan	270
South Korea	MHW South Korea	173
Japan	Ministry of Health, Labor and Welfare Japan	155
China	863 Program China	147
Japan	Society for the Promotion of Science Japan	126
China	Fundamental Research Funds for the Central Universities China	123

Abbreviations: NIH, National Institutes of Health; NCI, National Cancer Institute; MHW, Ministry of Health and Welfare.

### Analysis of Research Areas and Keywords

The most assigned subject category according to the WoS categories was *Oncology,* and therefore, it represents the most focused research area of this study (n = 14 436). The assignments of the following areas were very close with n = 6423 articles (*Surgery*) and n = 6329 (*Gastroenterology* & *Hepatology*). Subsequently, *General and Internal Medicine* followed with n = 2285 articles and *Pathology* with n = 2174.

**Figure 8:**
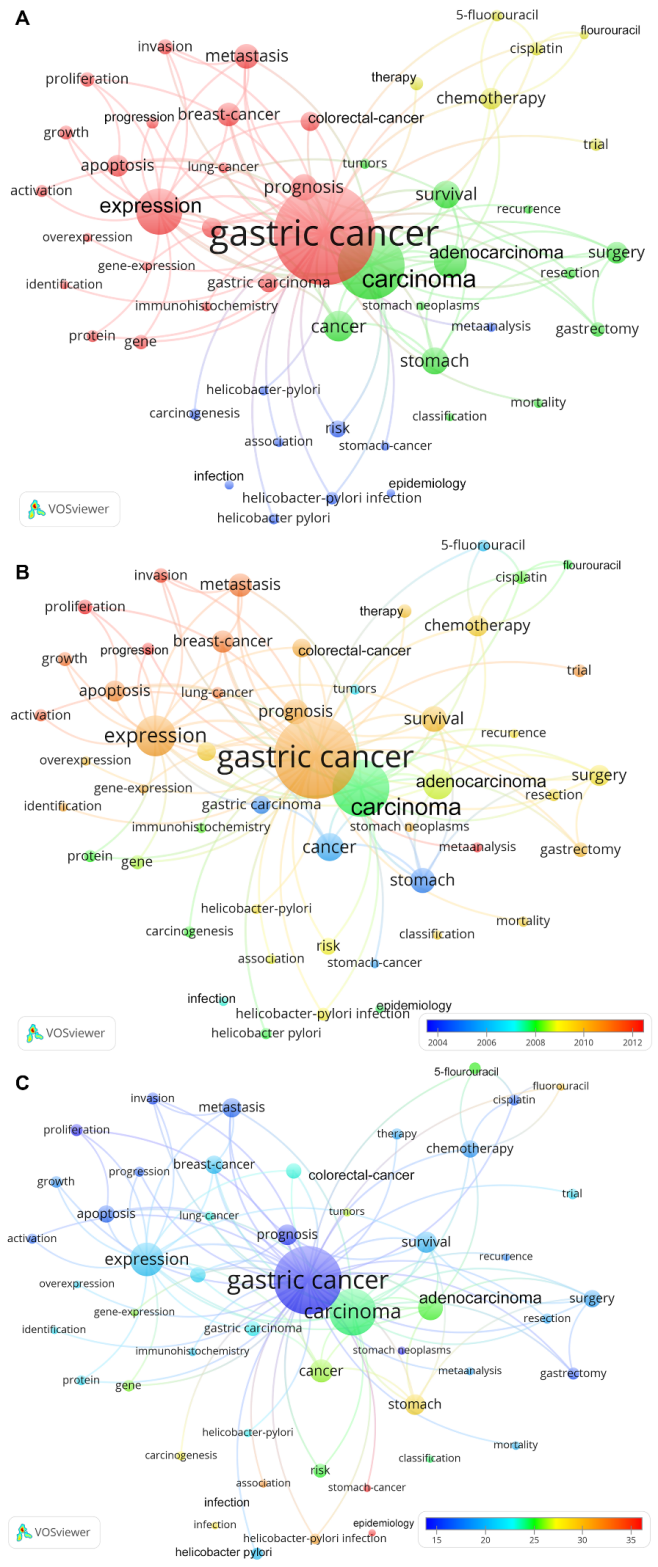
Author’s Keywords. Threshold: occurrence of keywords >100 times. (A) Cluster analysis. (B) Chronological assignments. (C) Times cited.

The evaluation of the author’s keywords occurring at least 100 times (threshold) showed four different clusters. One cluster represents issues referring to genetics, and tumor growth aspects. Another one comprises articles on pharmaceutical therapy forms. Surgical approaches are focused in a separate cluster. Epidemiological aspects and the association to *H. pylori* infection are dealt with in the last cluster of keywords (Figure 8A).

Furthermore, the keywords were analyzed regarding the time of their occurrence (Figure 8B). In this respect, the first cluster of genetics and cancer development seems to be the most current. Also striking are meta-analyses, which are also of recent date.

Another focus was laid on the evaluation of the number of citations each keyword received. Resulting, the keyword “epidemiology” can be singled out as the most cited (Figure 8C).

## Discussion

### Methodological Limitations and Strengths

The most cited paper from Finland by Lauren et al^22^ could not be assigned to a country of origin by the software used. Due to the evaluation frame from 1973 onwards, it could not be included in the geographic analysis of the citation rates. Prior to this date, the articles are seldom assigned to a country of origin, so that the application of the algorithm was not feasible. With a manually calculated value, Finland would be pushed to position 8 (cr = 56.66) before New Zealand. In retrospect, this individual value should not be manually integrated in the overall analysis, because the inclusion of one single value would endanger the objectivity and reproducibility of results. However, this example shows the limitations of the applied method. The accuracy of the results always depends on the completeness and the correctness of the entries. This problem also applies to the evaluation of citation-based parameters. Incorrect information or self-quotations distort the results. The analysis of funding sources covers only the clearly specified institutions or programs. Many grants are listed only by numbers or abbreviations, which could not be exactly determined. Additionally, unclear designation and information did not allow the evaluation of every funded article. Nevertheless, because of the big amount of data and the careful and unified application of the methodology, these effects can be dismissed and the database as well as the findings can be regarded as representative.

### Development of the Publication Output

The number of annual articles follows the usual pattern of scientific output and an exponential development can be shown. Also, the increase of the citation numbers ran steadily with the exception of some years with high prolific articles. In 1965, the most cited article from Pekka Laurén was published. It histologically classified GC for the first time into intestinal, diffuse and indeterminate types.^22^ This classification was outdated by the World Health Organization (WHO) classification of 2010 that distinguished GC according to the pattern of cells into tubular, mucinous, papillary or poorly cohesive types and uncommon variants.^36^ In 2010, the highest number of citations has been received so far. Due to the effect of the so-called cited half-life, publications that are published later are too new to achieve the maximum citation frequency. The cited half-life in biomedical research areas is proven to be about 8 years.^18,37^ It can therefore be assumed that the number of citations will continue to increase after 2010 as well in a later analysis.

Additionally, the articles published in 1991 got many citations. In this year, the association between *H. pylori* infection and GC stood in the focus of scientific interest. In 1982, Marshal and Warren identified a bacterium (at the time named *Campylobacter pyloridis*) that was present in all patients with gastritis and related it to the etiology.^38^ Later, this bacterium was reclassified as *H. pylori*. The first article in WoS that mentioned *H. pylori* was published in 1989. In 1990, its influence on GC was focused for the first time, but the first profound evidence was reported in three studies published in 1991.^23,24,39^ All three publications received more than 1000 citations and belong to the most cited studies of the database. The IARC classified *H. pylori* as a class I carcinogen in 1994.^40^ A positive effect of *H. pylori* eradication on the prevention of GC was found in a Japanese study from the year 2001. This year is also one of the years with the highest citation rate. Moreover, other therapeutic approaches were in focus in 2001, eg, the impact of chemoradiotherapy or endoscopic mucosal resection.^30,41^

The assumed positive effect of COX-2 selective nonsteroidal anti-inflammatory drugs^42^ led to an increase in publication output at the end of the 20th century. However, the risk of myocardial infarction as a consequence set an end to this enthusiasm shortly afterwards. In the present study, this influence is only slightly recognizable in the development of the publication numbers by the somewhat steeper rise in the 1990s and the flattened curve running afterwards (Figure 1). Nevertheless, the scientific interest is unbroken. It continues to increase exponentially and more strongly than in other cancer types. The citations numbers followed an equal development as their benchmark publications, with the exception of the years 1965, 1991, and 2001. Especially 1965 is emphasized in the findings of the chronological development of the citation rate, too. The interpretation of the citation rate emphasizes years with rather low publication volume and high citation numbers.

### Countries’ Publication Performance

In the United States, the expenditures for R&D, the scientific infrastructure and the conditions for researchers lead to an outstanding publication output that could be documented already in previous studies.^33,43^ Albeit this usually leading position, the publication numbers on GC does not show the United States as most publishing country. In this study, the United States reached only the third position.

With more than 8000 articles on GC, China and Japan were the most publishing countries in this analysis. Since the findings show that the number of articles is relatively closely linked to the countries’ expenditures on R&D, it can be assumed that the publication output of China on GC will rise even more in the future, because China is assumed to reach a top position regarding the R&D expenditures very soon. Actually, it could be ranked even higher, because the salaries of basic research scientists are not included. However, in terms of citation numbers China was falling behind the United States and Japan. The quality of Chinese research has often been called into question, but it is assumed to improve currently. A report from 2017 showed that, overall, Chinese scientific articles were the second most cited in absolute numbers, but the citation rate remains below the global average.^44^ Despite the high number of scientists, advanced graduates are as rare as the number of graduates returning from abroad. The incentive not only for the quantity but also for the quality of research must be strengthened.^45^ Together with South Korea, Japan, and China are the most affected countries and they were among the most publishing countries of the present study.

China’s and South Korea’s contribution over time was steadily increasing, whereas the number of Japanese articles had its maximum between 1993 and 1997 and decreased afterwards. In the early 1990s of the last century the evidence became clear that eating habits and environmental factors affect GC to a large extent and cause the regional differing incidence rates. Japanese children got GC more often, due to the eating habits that includes the consumption of raw fish and meat.^46^ Only in Japan the survival rate is relatively good with up to 90%.^3,4^ This may be a result of early diagnosis by regular check-ups. However, changes in the gastric mucosa are often diagnosed as GC in Japan. In this case, most other countries do not diagnose cancer, because the criteria for cancer of invasive growth is not met. This can bias the statistics especially the survival rates in Japan.^47^ The evaluation of the funding sources showed that the governments of China, the United States, Japan, and South Korea supported most GC research, leading to high-impact publications. Here, the highly affected countries fulfilled their obligation and the United States lived up to their leading status. The comparison of the funded articles’ citation rates revealed likewise the huge resonance of the USA/NIH funds. The growing Chinese citation numbers can only partly be explained by the increasing funding. It is also due to a shift towards prolific research field, eg, cancer biology. Also, international collaborations are causing the rise in citation numbers.^44^ Studies found the best science as an outcome of international networks and that collaboration articles are cited more often.^48,49^ In our study, the most publishing countries China, Japan, the United States, and South Korea were also the most collaborating countries, with the United States at its core. It has already been shown that the number of collaborations between the United States and Asian countries is increasing sharply.^49^ China has built up a strong international network with more than 150 countries since its economic reform and open-door policy. Additionally, it has been found that the number of collaboration articles in China rose above average.^50^

It is explainable that the socio-economic status and the expenses for R&D and political strategies have strong influences on the countries’ publication output. Additionally, the importance of epidemiological challenges for science is clear. Here, the association between the incidence rates of the countries and the publication endeavors is also given. Nevertheless, low-income countries with an overload of disease anamneses were only partially involved in GC research. Africa in particular was often underrepresented. African research has been proved to be more local than international, in addition to other disadvantages based on socio-economic indicators. The industrial countries keep privileges that result in a negative self-perception of African scholars.^51^ Hence, the international network of GC research is still mainly dominated by industrial countries. After all, the growing participation of emerging economies is changing the global balance affecting new ways of scientific working. Due to cross-border health hazards and priorities, international approaches will continue to gain weight.^49^ Especially for low economies, the advantage of collaborations lies in the “accumulation of knowledge,” which enables important economic growth through the exchange of skills and techniques.^52^ It has been found that the number of publications correlates with the number of international collaborations and with the impact of the publications.^53,54^

## Conclusion

The impact of the status-quo of scientific infrastructure standards in combination with political frameworks becomes apparent when looking at the global publication output and the countries’ funding behavior for GC research. The association of the publication endeavors with the epidemiological burden is also noticeable, albeit to a far lesser extent. Especially, the highly affected low-economy countries are under-represented. Therefore, the encouragement of scientists from low-income countries and the respectful exchange and engagement are on demand by developing advantageous partnerships. Thus, the exchange of knowledge and experience between countries of different cultural backgrounds and scientific infrastructures should be enforced for mutual benefit. Although both geographical and cultural proximity play a role in international networking, the strongest cooperation in GC has been between the United States and the Asian countries China, Japan, and South Korea.

The growing incidence of GC in the industrial world suggests that the future epidemiological data will be different than current rates. This trend will lead to regional peculiarities that differ from those of today and which must be taken into account by adapting scientific priorities. Only by the profound insight of the successes and failures of past, current and expected research efforts a targeted planning for new strategies can be realized.

## Ethical issues

There is no human or animal respectively their tissues or cells involved in this study. Therefore, no vote was necessary.

## Competing interests

Authors declare that they have no competing interests.

## Authors’ contributions

DK and DAG contributed to conception and design. DK, MB, NS, DB, and DAG contributed to the analyses, the interpretation of data and the draft of the article. DK wrote the article. DK, MB, NS, DB, and DAG revised the article. DK, MB, NS, DB, and DAG have participated in the final approve of the manuscript.

## Supplementary files

Supplementary file 1 contains Tables S1 and S2.

## Key Messages

**Implications for policy makers**

- Regarding the publication output, the allocation of high-income countries on higher ranks and that of middle-income countries at lower positions could be pointed out. Low-income countries showed no relevance at all.
- Wide international collaborations have shown to be successful, as shown by the growth of citation numbers. This should be considered while planning future approaches.
- Against the backdrop of the changing epidemiological situation, all countries are called upon to contribute according to their economical and scientific possibilities.
- An extensive network for gastric cancer (GC) research is crucial to meet future challenges worldwide.
- The findings can help to plan and establish such a global network that includes high infrastructure countries and most affected and less developed regions alike to satisfy the differing geographical backgrounds and possibilities.

**Implications for public**

Currently, gastric cancer (GC) is ranked third as cause for cancer death worldwide, whereby two-thirds occur in low-income countries. The epidemiological statistic is changing over time, so that new challenges have to be meet in many countries. In contrast to the statistics so far, a new GC type known as CYF (corpus-, young age, female)-cancer affects mainly less-poor regions, as well as young and female persons. The correlation of research and development (R&D) expenditures with the efforts of the countries in GC research shows the influence of the national status quo of scientific infrastructure standards in addition to the political framework conditions. The association of the publication endeavors with the epidemiological burden is also noticeable, albeit to a far lesser extent. Here, the highly affected countries of the low-income world are mostly extremely under-represented according to their publication output.
