# Supplementary material for: Gastric Cancer: Bibliometric Analysis of Epidemiological, Geographical and Socio-Economic Parameters of the Global Research Landscape
Source: Int J Health Policy Manag. 2020 Mar 1;10(3):118–28. doi: 10.34172/ijhpm.2020.29 (PMC7947903; doi:10.34172/ijhpm.2020.29)
Supplement: Supplementary file 1 — contains Tables S1 and S2. [file ijhpm-10-118-Supp1.pdf]

Supplementary file 1

Table S1. Socioeconomic parameters of countries with more than 30 articles on gastric cancer

Country	No. of Articles	Rank 1 (WB Economies)	R_{POP}	Rank 2 (WB Economies)	R_{GDP}	Rank 3 (WB Economies)
China	8931	1 (UMI 1)	6.50	32 (UMI 1)	419.89	17 (UMI 1)
Japan	8454	2 (HI 1)	66.72	2 (HI 2)	1714.11	2 (HI 2)
United States	4051	3 (HI 2)	12.50	25 (HI 25)	218.27	33 (HI 29)
South Korea	3424	4 (HI 3)	67.24	1 (HI 1)	1775.01	1 (HI 1)
Germany	1750	5 (HI 4)	21.68	15 (HI 15)	439.81	16 (HI 16)
Italy	1427	6 (HI 5)	23.01	14 (HI 14)	642.50	10 (HI 10)
United Kingdom	1045	7 (HI 6)	16.22	18 (HI 18)	374.82	20 (HI 19)
Taiwan	732	8 (HI 7)	31.20	7 (HI 7)	650.67	9 (HI 9)
France	686	9 (HI 8)	10.26	27 (HI 27)	250.64	30 (HI 26)
Spain	569	10 (HI 9)	11.72	26 (HI 26)	336.69	22 (HI 21)
Turkey	507	11 (UMI 2)	6.32	33 (UMI 2)	303.59	28 (UMI 3)
Russia	494	12 (UMI 3)	3.47	36 (UMI 4)	131.91	39 (UMI 6)
The Netherlands	488	13 (HI 10)	28.68	10 (HI 10)	563.58	13 (HI 13)
Sweden	351	14 (HI 11)	35.52	5 (HI 5)	704.68	8 (HI 8)
Canada	339	15 (HI 12)	9.59	28 (HI 28)	202.51	34 (HI 30)
Portugal	309	16 (HI 13)	28.52	11 (HI 11)	1040.05	3 (HI 3)
Australia	295	17 (HI 14)	12.83	23 (HI 23)	248.11	31 (HI 27)
Poland	292	18 (HI 15)	7.58	30 (HI 30)	277.57	29 (HI 25)
Iran	284	19 (UMI 4)	3.43	37 (UMI 5)	194.65	36 (UMI 4)
Brazil	272	20 (UMI 5)	1.32	41 (UMI 8)	86.76	42 (UMI 9)
India	240	21 (LMI 1)	0.19	47 (LMI 2)	27.52	46 (LMI 2)

Finland	239	22 (HI 16)	43.47	3 (HI 3)	999.16	4 (HI 4)
Greece	232	23 (HI 17)	21,53	16 (HI 16)	798.62	6 (HI 6)
Singapore	231	24 (HI 18)	39.95	4 (HI 4)	474.43	15 (HI 15)
Switzerland	191	25 (HI 19)	23.35	13 (HI 13)	386.41	18 (HI 17)
Norway	186	26 (HI 20)	35.33	6 (HI 6)	510.01	14 (HI 14)
Chile	167	27 (HI 21)	9.46	29 (HI 29)	382.94	19 (HI 18)
Denmark	163	28 (HI 22)	29.14	9 (HI 9)	615.56	12 (HI 12)
Belgium	156	29 (HI 23)	13.67	20 (HI 20)	306.72	27 (HI 24)
Austria	138	30 (HI 24)	15.84	19 (HI 19)	331.81	24 (HI 22)
Mexico	126	31 (UMI 6)	1.02	44 (UMI 11)	54.62	44 (UMI 11)
Ukraine	112	32 (LMI 2)	2.53	39 (LMI 1)	320.18	26 (LMI 1)
Israel	106	33 (HI 25)	12.97	22 (HI 22)	356.90	21 (HI 20)
Colombia	83	34 (UMI 7)	1.76	40 (UMI 7)	120.22	40 (UMI 7)
Romania	74	35 (UMI 8)	3,.43	38 (UMI 6)	167.80	37 (UMI 5)
Hungary	65	36 (HI 26)	6.58	31 (HI 31)	242.90	32 (HI 28)
Ireland	65	37 (HI 27)	13.12	21 (HI 21)	200.43	35 (HI 31)
South Africa	65	38 (UMI 9)	1.20	43 (UMI 10)	88.28	41 (UMI 8)
New Zealand	56	39 (HI 28)	12.52	24 (HI 24)	320.37	25 (HI 23)
Slovenia	50	40 (HI 29)	25.28	12 (HI 12)	756.09	7 (HI 7)
Czech Republic	47	41 (HI 30)	4.42	35 (HI 32)	133.94	38 (HI 32)
Estonia	38	42 (HI 31)	30.19	8 (HI 8)	981.91	5 (HI 5)
Venezuela	38	43 (UMI 10)	1.23	42 (UMI 9)	81.09	43 (UMI 10)
Serbia	34	44 (UMI 11)	4.76	34 (UMI 3)	334.98	23 (UMI 2)
Latvia	32	45 (HI 32)	16.28	17 (HI 17)	629.05	11 (HI 11)
Argentina	31	46 (HI 33)	0.71	45 (HI 33)	35.25	45 (HI 33)
Thailand	31	47 (UMI 12)	0.45	46 (UMI 12)	26.70	47 (UMI 12)

HI = high-income countries, UMI = upper-middle-income countries, LMI = lower-middle-income countries (World Bank classification: https://datahelpdesk.worldbank.org/knowledgebase/articles/906519#High_income).

Table S2. Comparison of OECD (*Organization for Economic Co-operation and Development*) countries, sorted by incidence rate,[15] R&D = Research & Development[32]

**OECD Country**	**No. of Articles**	**Incidence Per 100 000 People**	**R&D Expenditures in Mill. US-Dollars**
South Korea	3424	39.6	73.59
Japan	8454	27.5	154.71
China	8931	20.7	376.90
Chile	167	17.8	1.44
Russia	494	13.3	37.32
Latvia	32	12.9	0.27
Turkey	507	12.5	15.64

Estonia	38	11.4	0.51
Portugal	309	11.0	3.44
Slovakia	11	10.7	1.79
Hungary	65	9.4	3.24
Slovenia	50	8.9	1.27
Poland	292	8.3	9.34
Romania	74	8.3	1.92
Ireland	65	7.5	3.36
Italy	1427	7.2	27.06
Germany	1750	6.7	101.58
Spain	569	6.6	17.98
Greece	232	6.5	2.48
Argentina	31	6.4	5.11
Belgium	156	5.9	11.32
Czech Republic	47	5.8	6.08
Mexico	126	5.6	10.63
Luxembourg	6	5.5	0.64

Switzerland	191	5.4	15.22
Austria	138	5.3	11.29
Israel	106	5.2	11.16
France	686	4.9	55.79
Australia	295	4.6	20.37
The Netherlands	488	4.5	15.44
New Zealand	56	4.5	1.98
Denmark	163	4.2	7.47
Finland	239	4.2	6.01
Norway	186	4.1	5.79
United States	4051	4.1	456.90
Canada	339	4.0	25.08
South Africa	65	4.0	5.35
United Kingdom	1045	3.9	41.78
Iceland	18	3.6	0.31
Sweden	351	3.3	14.17
